# Using Directional Deep Brain Stimulation to Co-activate the Subthalamic Nucleus and Zona Incerta for Overlapping Essential Tremor/Parkinson's Disease Symptoms

**DOI:** 10.3389/fneur.2018.00544

**Published:** 2018-07-05

**Authors:** Ramsey A. Falconer, Sean L. Rogers, Mahesh Shenai

**Affiliations:** Inova Parkinson's and Movement Disorders Center, Falls Church, VA, United States

**Keywords:** deep brain stimulation, directional deep brain stimulation, current steering, parkinson disease, essential tremor, mixed tremor, axially asymmetric stimulation, co-activating targets

## Abstract

This index case report describes a novel programming approach that utilizes the 8-contact directional Deep Brain Stimulation (DBS) lead to effectively control the akinesia, rigidity and tremor of Parkinson's Disease (PD), as well as a severe kinetic tremor of Essential Tremor (ET), in a patient with overlapping symptoms of both PD and ET. Through utilizing a bipolar directional montage on a single segmented contact, symptom control was attained via likely co-activation of the Subthalamic Nucleus (STN) and the adjacent Zona Incerta (ZI). The patient is a 67-year-old professional guitarist with a long-standing diagnosis of ET manifesting with bilateral kinetic tremor, who then developed right lateralizing symptoms indicative of PD. After optimal medical management did not confer sufficient control, he underwent left-sided unilateral DBS targeting the STN. Both intraoperatively and post-operatively, omnidirectional, and directional electrode review resulted in significant akinesia, rigidity, and as well as resting tremor control but failed to sufficiently improve the kinetic tremor. As electrode 2B was shown to be the most efficacious with the largest therapeutic window, a bipolar directional montage on a single segmented contact was tried with the idea of possibly further extending the axial asymmetry of the directional stimulation toward the adjacent ZI to impact the kinetic tremor. This montage resulted in full kinetic and resting tremor control as well as akinesia and rigidity response [2B cathode (–), 2A anode (+), 2C anode (+) (1.4 mA, rate 160 Hz, pulse width 60 μs)]. At 6 months post initial programming, no montage changes have been made, and the patient has experienced a reduction in Motor UPDRS scores from 23 to 3 (evaluated off medication), full resolution of kinetic tremor and normalization of handwriting, as well as significant reduction in his medication requirements. This patient's response to a single segment bipolar directional montage, and lack of response from monopolar directional stimulation in the same area, does suggest the possibility of further axial asymmetric tissue activation and thus co-activation of both the dorsal STN and adjacent ZI. Further modeling and study are warranted.

## Introduction

**Figure 1:**
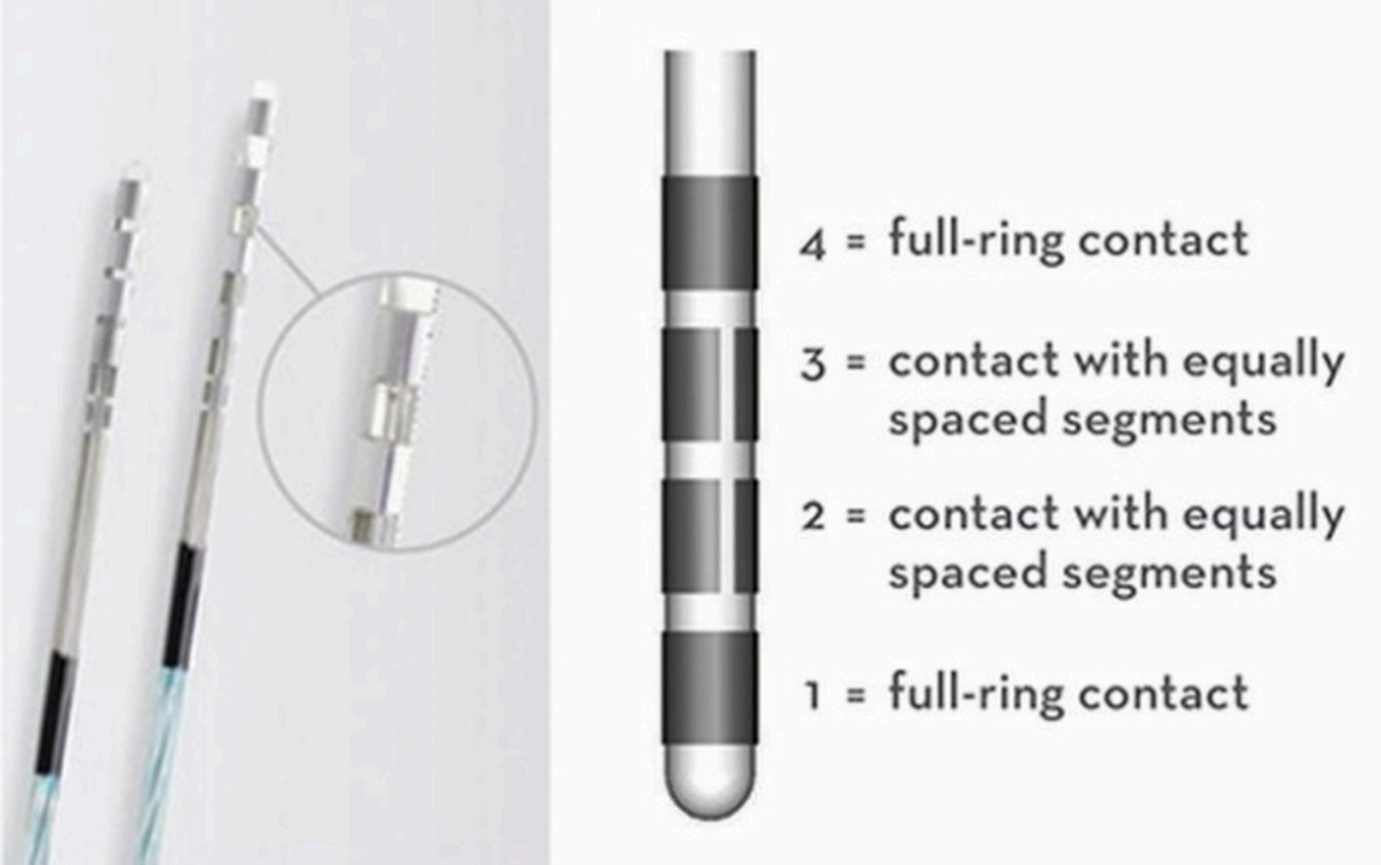
Abbott^TM^ 8-segmented directional DBS lead, showing the 4 numbered leads (1–4) and how leads 2 and 3 are divided into 3 equally spaced segments (~90° each), allowing for directional stimulation.

The advent of directional Deep Brain Stimulation (DBS) has expanded the possibilities for both simple and complex field shaping, as well as given a theoretical potential to effectively activate two targets simultaneously with one lead. With the classic 4-contact ring electrode that lacked directional lead segmentation, programming is approached through successively elevating levels of programming complexity with the goal of increasingly elaborate ways to restrict the field of energy delivered, activating the intended circuits but not those which would drive a side-effect. Be it double monopolar or bipolar montages, or even more complex field fragmentation in time via interleaving, these programming permutations are limited by being axially symmetric to the axis of the lead. The current 8-contact directional DBS lead allows for stimulation to be delivered in an axially asymmetric fashion, creating novel approaches to programming and improving therapeutic flexibility (Figure 1).

The effectiveness of directional stimulation has been shown to be on par with omnidirectional stimulation, with several advantages identified, including the ability to utilize a larger therapeutic window between symptom response and side effect. Furthermore, studies have shown that this wider therapeutic range can be achieved with a lower current threshold (1). These two separate points of improvement over omnidirectional can likely be attributed to the combination of a smaller surface area of the directional contact creating a higher density of energy and thus a larger and more effective volume of tissue activation (VTA) for the power used, as well as the ability to deliver the energy in an axially asymmetric way toward the intended area of activation. The benefits described are achievable while still maintaining the long-term symptom response and medication reduction expected from omnidirectional stimulation (2). Additionally, these benefits appear to be achievable without sacrificing battery or predicted device lifespan (3).

These properties of directional DBS create an effective tool in the DBS programmer's algorithm to utilize axially asymmetric field restriction and creates new avenues to explore novel approaches to stimulation. Case reports are starting to appear in the literature where the axially asymmetric stimulation was utilized to expand the therapeutic window and avoid identified side effects (4). It would therefore not be a large theoretical leap to utilize strategic current steering to co-activate two separate targets of anatomically adjacent structures.

Through this case report, we describe a novel programming approach that utilizes the 8-contact directional DBS lead to effectively control the akinesia, rigidity, and tremor of PD, as well as a severe kinetic tremor of ET, in a patient with overlapping symptoms of both PD and ET. In a subset of patients, an overlapping symptomatology has been identified that spans both disorders, suggesting a mixed disorder syndrome (5). The novel programming montage described here was discovered through successive sub-optimal total symptom response with the more classic approaches to programming, resulting in a montage that theoretically co-activates the dorsal aspect of the Subthalamic Nucleus (STN) as well as the Zona Incerta (ZI).

Regarding activation of the ZI for tremor control, several investigators have shown reduced tremor activity in both ET and PD (6–10). In 2016, a comprehensive literature review on posterior subthalamic area/caudal ZI stimulation concluded that stimulation of this area is a safe and effective treatment for refractory and atypical tremor (10).

## Case report

**Figure 2:**
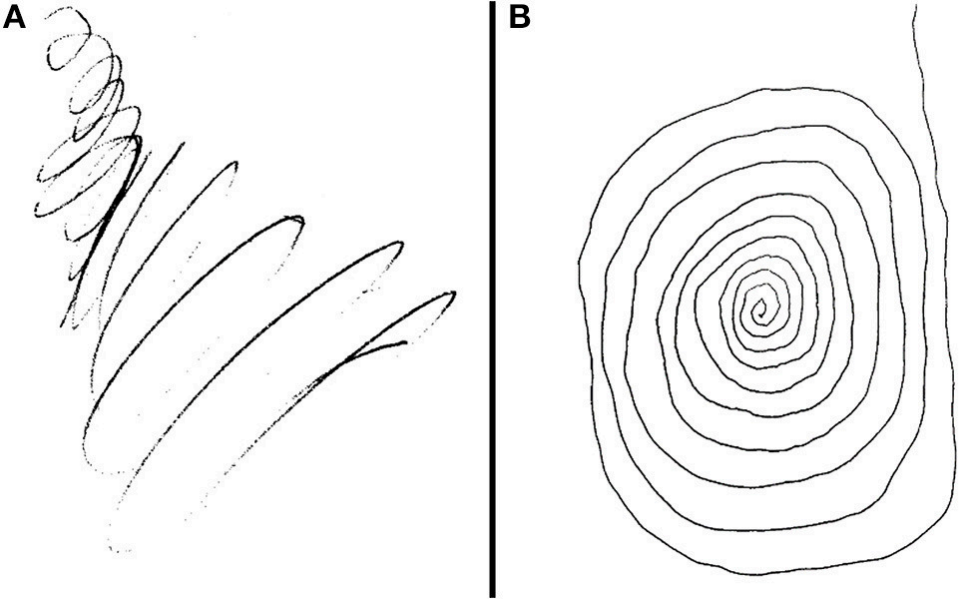
**(A,B)** Patient's attempt at drawing a spiral prior to DBS implantation and after implantation with activation of the following parameter: 2B (–), 2A (+), 2C (+). This is theoretically further extending stimulation into the adjacent ZI as well as maintaining stimulation in the STN.

The patient is a 67-year-old professional guitarist with a long-standing diagnosis of ET, diagnosed by the presence of a mild bilateral tremor with activity that would manifest with handwriting or tool use, as well as a familial inheritance pattern. Then, in his early 60 s, he developed a resting component to the tremor in his right arm as well as right-predominant bradykinesia and rigidity. He also began to experience gait changes and several nonmotor symptoms suggestive of a dopamine deficiency (hypophonia, constipation, REM-Behavioral Disorder, reduced sense of smell, and more). In addition to his pre-existing diagnosis of ET, he was given a diagnosis of PD after a significant symptomatic response to Levodopa therapy. His symptoms also began to severely impact his guitar playing ability and handwriting (Figure 2A).

He did not tolerate primidone, showed a sub-optimal response to propranolol, and either experienced side-effects or a sub-optimal akinesia and rigidity response to a combination pramipexole ER, rasagiline, and Carbidopa/Levodopa. The decision was made to pursue unilateral DBS implantation given the right predominance of his symptoms. STN was selected due to the predominance of akinesia and rigidity in addition to tremors.

He underwent stereotactic frame-based awake DBS implantation that utilized direct targeting technique on a pre-operative T2 FLAIR sequence, merged with intraoperative CT scan. The trajectory endpoint was target to the left dorsolateral STN at the anterior-posterior position of the anterior edge of the red nucleus, and at a lateral position 3 mm lateral to the most lateral point of the red nucleus. Microelectrode recordings were used to delineate the dorsal and ventral border of the STN, with macrostimulation confirming full rigidity and resting tremor resolution without adverse effect. An Abbott™ 8-contact directional lead was then placed. Intraoperative fluoroscopy was utilized to confirm accurate lead rotation with “hourglass” stereotactic marker ensuring the A contacts were placed in an anterior direction.

**Figure 3:**
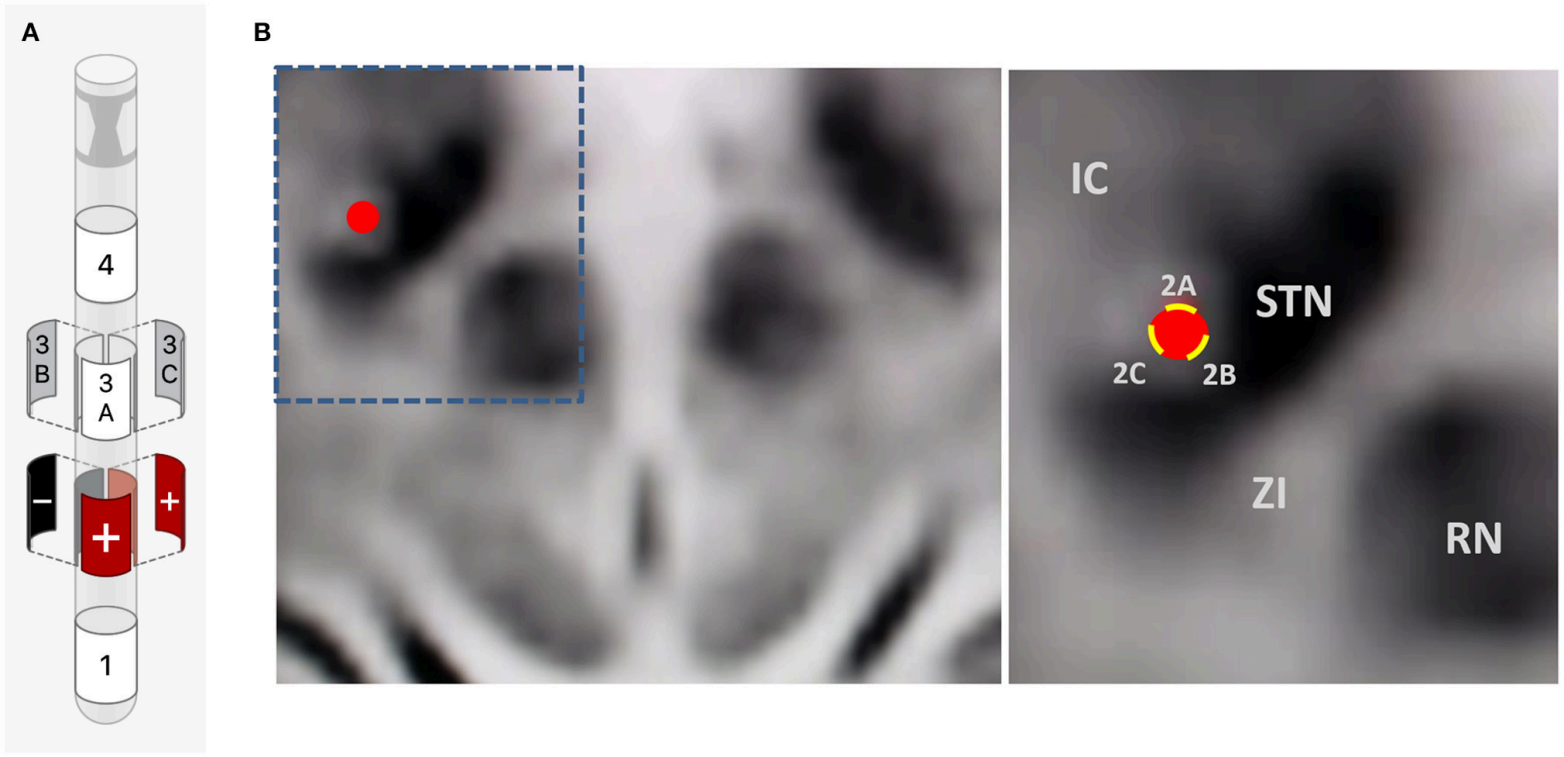
**(A)** Final bipolar directional montage on a single segmented contact: 2B cathode (–), 2A anode (+), 2C anode (+) (1.4 mA, rate 160 Hz, pulse width 60 μs). **(B)** Intraoperative imaging showing lead position and adjacent anatomy. IC, internal capsule; STN, Subthalamic Nucleus; ZI, Zona Incerta; RN, Red Nucleus; Red circle, representation of lead position; Yellow bars, representation of directional contacts.

Intraoperative testing at the deepest contact, Contact 1 as cathode (–) and Case as anode (+), showed nearly complete akinesia/rigidity response, resting tremor resolution, and fine motor normalization at 1.5 mA. His action tremor persisted though, with an inability to write a spiral on testing. This was repeated at the highest contact, Contact 4, again showing full parkinsonian symptom response but the kinetic tremor and handwriting component persisted. Contact 2B and then 3B were activated as cathode (–) to take advantage of axially asymmetric tissue activation, shifting stimulation theoretically in a posterior-medial plane in the direction of the ZI. The action-oriented tremor continued, with minimal change to severe kinetic tremor with handwriting. Next, the montage 2B cathode (–), 2A anode (+), 2C anode (+) was tried, envisioning a “tear-drop” shaped stimulation, activating the area adjacent to the bore of the electrode on the 2 level given anode activation (STN), and stimulation extending posterior and medial through the cathode activation at 2B, activating the ZI (Figures 3A,B). With this montage, he experienced full akinesia and rigidity control as well as both resting and kinetic tremor arrest. His writing spirals normalized (Figure 2B) and he was able to play guitar unencumbered in the operating room.

On outpatient programming 4 weeks later, a stepwise monopolar and directional electrode review was performed resulting in significant reduction in akinesia and rigidity as well as resting tremor, but the kinetic tremor continued to affect his handwriting and guitar skills. Even the 2B or 3B directional electrode as monopolar cathode (–) failed to show full kinetic tremor response up to 3 mA. The 2B cathode monopolar review showed the best parkinsonian symptom response with the largest therapeutic window. Considering the possibility of single contact bipolar directional stimulation, we then attempted and found full kinetic tremor resolution as well as normalization of his right lateralizing akinesia and rigidity and resting tremor at the montage 2B cathode (–), 2A anode (+), 2C anode (+) (1.4 mA, rate 160 Hz, pulse width 60 μs) (Figures 3A,B). At 6-month follow-up, he continued to experience full kinetic and resting tremor control, as well as reduction in his Motor UPDRS scores from preop 23 to 3, both recorded off medication. He also was able to discontinue propranolol as well as pramipexole ER and reduce his other Parkinson's medications to only rasagiline 1 mg daily and Rytary^TM^ (Carbidopa/Levodopa ER) 23.75/95 mg 3x daily. Furthermore, he has not needed an adjustment to his original programming montage 6 months out from implantation. He is also back to playing guitar professionally.

## Discussion/conclusion

There exists a subset of patients experiencing the overlapping symptomatology of both PD and ET, presenting with akinesia, rigidity, and resting tremor in addition to a severe kinetic tremor. These patients present a challenge when utilizing DBS, as different targets can be utilized to impact different symptoms, be it VIM for tremor control or STN for impact on akinesia, rigidity and a component of tremor suppression (5, 11–14).

In this index case report, we report the possible co-activation of the dorsal aspect of the STN and the adjacent ZI through the utilization of a bipolar directional montage on a single segmented contact. In this patient, a kinetic tremor response was not achieved on monopolar or directional contact review in the accurately placed STN lead, suggesting the possibility of further VTA extension by placing anode and cathode on different contacts of the same directional contact [2B cathode (–), 2A anode (+), 2C anode (+)] (Figures 3A,B). This theoretically further extended the axially asymmetric VTA of the 2B directional contact posteriorly and medially, activating both structures.

This raises an interesting question as to the field restriction created and the extent of the VTA impacted by segmented bipolar directional stimulation. Does setting a directional contact into bipolar stimulation create further axially asymmetric stimulation given the interplay between anode and cathode? It does seem via our patient's symptom response that this montage is likely activating the adjacent ZI in addition to the dorsal aspect of the STN.

While an exciting possibility given the new theoretical stimulation paradigms allowed by directional DBS leads, this case report is limited as being one patient, and further VTA modeling and study is warranted, especially given the likely impact on battery life with these stimulation parameters. That being said, the possibility of co-activating adjacent structures through directional stimulation could open treatment options for overlapping tremor/akinesia/rigidity (PD/ET) patients.

This index case report was published with the written informed consent of the patient.

## Ethics statement

This study was a case report of a standard surgical procedure, which stimulation was applied within the bounds of accepted practice. The patient gave full consent to report his case.

## Author contributions

RF lead author. SR and MS contributing author.

### Conflict of interest statement

The authors declare that the research was conducted in the absence of any commercial or financial relationships that could be construed as a potential conflict of interest.
